# The fice course and qualification - experience from the cheshire and merseyside group

**DOI:** 10.1186/2197-425X-3-S1-A610

**Published:** 2015-10-01

**Authors:** J Dudziak, N Bolton, G McNeil, V Paul, A Raithatha, G Rogers, K Booker, E Lima, P Jeanrenaud, K Sim, C Wong, A Mohammed, V Mahendran, I Welters, A Tridente

**Affiliations:** Whiston Hospital, Liverpool, United Kingdom; ICU, Nottingham, United Kingdom; STH, Sheffield, United Kingdom; Royal Liverpool University Hospital, Liverpool, United Kingdom; Sheffield University, Infection and Immunity, Sheffield, United Kingdom

## Introduction

The utility of echocardiography for assessing the critically ill patient has long been recognised. The Focused Intensive Care Echocardiography (FICE) training pathway is endorsed by the Intensive Care Society of the UK. Candidates attend a full day course consisting of lectures and practical sessions. Subsequently, every candidate is required to complete a logbook of 10 supervised cases and 40 further cases within 12 months. The qualification is attained by passing a triggered assessment.

## Objectives

We aimed to establish the effects of implementing the first part of the FICE training programme in our region.

## Methods

A regional FICE course was established and run on two occasions. All sessions were delivered by British Society of Echocardiography accredited consultants and echo-sonographers. A course evaluation and a survey of practice were completed, allowing candidates to rate their confidence in the FICE curriculum items before and after the course.

## Results

A total of 28 candidates gave their rating. The grades of doctors attending were as follows: 12 (42.8%) consultants 12 (42.8%) specialty registrar trainees and 4 (14.4%) more junior medical trainees. On a scale of 1 to 5, all components of the course scored highly. Candidates particularly liked the practical (median rating 5, IQR=interquartile range 5-5) and the anatomy on ultrasound (median rating 5, IQR 4-5) sessions. Previous echo experience was limited: 12 (42.9%) candidates reported no previous experience, 8 (28.6%) reported 10 previous scans or more; no candidate had performed more than 40 echocardiograms. Across all domains surveyed, candidates' self-rated competence (on a scale of 1 to 5) rose by 1 point or more. The course especially improved the self-reported familiarity with echo anatomy (median pre-course 2, IQR 2-3 to post-course median 4, IQR 4-5 score), understanding of machine optimisation (pre 2, IQR 1-3 to post 4, IQR 3-4) and practical echo skills (pre 1, IQR 1-2 to post 3, IQR 2-4).

## Conclusions

The FICE course is rated highly for its hands-on approach and improves candidates' confidence and self-rated knowledge in the important domains of the FICE curriculum. The feasibility of attaining the qualification by completing a 50 case logbook within 12 months will be the subject of further evaluation.

